
JOURNAL INFORMATION
==============================
NLM Title Abbreviation: Wirtschaftsdienst
Journal ID: Wirtschaftsdienst
NLM Journal ID: 101086612

Wirtschaftsdienst (Hamburg, Germany : 1949)

ISSN: 0043-6275

ARTICLE INFORMATION
==============================
PMCID: PMC7237609
PMID: 32454543
DOI: 10.1007/s10273-020-2651-1
Article ID: 2651
Article version: 1
Subjects: Zeitgespräch

WTO — die Hüterin des Welthandels in der Krise

Mildner Stormy-Annika S.Mildner@bdi.eu

von Unger Eckart E.vonUnger@bdi.eu

Tepper Katherine K.Tepper@bdi.eu

grid.469691.5 0000 0001 0465 2215 Abteilung Außenwirtschaftspolitik, Bundesverband der Deutschen Industrie e.V., Breite Straße 29, 10178 Berlin, Deutschland
Electronic publication date: 2020 May 20
Print publication date: 2020
Volume: 100
Issue: 5
First page: 332
Last page: 337
Copyright: © Der/die Autor(en) 2020
License: Open Access Dieser Artikel wird unter der Creative Commons Namensnennung 4.0 International Lizenz veröffentlicht, welche die Nutzung, Vervielfältigung, Bearbeitung, Verbreitung und Wiedergabe in jeglichem Medium und Format erlaubt, sofern Sie den/die ursprünglichen Autor(en) und die Quelle ordnungsgemäß nennen, einen Link zur Creative Commons Lizenz beifügen und angeben, ob Änderungen vorgenommen wurden.
Die in diesem Artikel enthaltenen Bilder und sonstiges Drittmaterial unterliegen ebenfalls der genannten Creative Commons Lizenz, sofern sich aus der Abbildungslegende nichts anderes ergibt. Sofern das betreffende Material nicht unter der genannten Creative Commons Lizenz steht und die betreffende Handlung nicht nach gesetzlichen Vorschriften erlaubt ist, ist für die oben aufgeführten Weiterverwendungen des Materials die Einwilligung des jeweiligen Rechteinhabers einzuholen.
Weitere Details zur Lizenz entnehmen Sie bitte der Lizenzinformation auf http://creativecommons.org/licenses/by/4.0/deed.de.
License URL: https://creativecommons.org/licenses/by/4.0/

issue-copyright-statement: © The Author(s) 2020

==============================
Dr. Stormy-Annika Mildner leitet die Abteilung Außenwirtschaftspolitik des Bundesverbands der deutschen Industrie (BDI) und ist Adjunct Lecturer an der Hertie School of Governance in Berlin.

Der wirtschaftliche Einschnitt durch die COVID-19-Pandemie ist massiv. In Deutschland und Europa, aber auch weltweit musste eine Vielzahl von Unternehmen ihre Produktion weitestgehend einstellen. Die meisten Geschäfte sowie kulturelle Einrichtungen mussten schließen. Dies führte zu einem massiven Einbruch der Wirtschaftsleistung. Internationale Wertschöpfungs- und Lieferketten wurden unterbrochen; internationale Absatzmärkte brachen weg. Es überrascht daher nicht, dass der Internationale Währungsfonds (IMF) in seinem jüngsten World Economic Outlook von Mitte April 2020 davon ausgeht, dass die Weltwirtschaft im laufenden Jahr um 3 % schrumpfen wird (IMF, 2020a). Noch im Januar 2020 hatte der IWF für dieses Jahr ein globales BIP-Wachstum von 3,3 % prognostiziert (IMF, 2020b). Sollte sich die Corona-Pandemie noch in diesem Jahr abschwächen, rechnet der IWF mit einer teilweisen Erholung der Weltwirtschaft im Jahr 2021.

Auch die Prognosen für den Welthandel sind düster. Die Welthandelsorganisation (WTO) rechnet mit einem Rückgang des Warenhandels von 13 % bis 32 % in diesem Jahr, je nach Länge der Eindämmungsphasen in den großen Volkswirtschaften (WTO, 2020c). Neben den Produktionsausfällen stören zunehmend auch nationale Ausfuhrbeschränkungen den Handel. Just-in-time-Produktion ist in der derzeitigen Lage unmöglich geworden. Lagerbestände entstehen, wo es sie bislang kaum oder gar nicht gab. Unternehmen müssen besonders in der Logistik ihre Aktivitäten immer mehr einschränken (Obstfeld und Posen, 2020).

Aufgrund des asymmetrischen Krisenverlaufs dürften sich Welthandel und weltweite Investitionsströme nur langsam wieder erholen. Anders als in der jüngsten Finanz- und Wirtschaftskrise 2008 bis 2011 sollte auch nicht damit gerechnet werden, dass die Schwellen- und Entwicklungsländer als Wachstumslokomotiven exportabhängige Länder wie Deutschland schnell wieder aus der Krise ziehen werden. Denn auch sie leiden massiv unter der Krise.

Die Corona-Epidemie kommt für das multilaterale Handelssystem zu einem denkbar schlechten Zeitpunkt. Während die WTO zusammen mit ihrem Vorgänger, dem Allgemeinen Zoll- und Handelsabkommen (GATT), über Jahrzehnte zu fairen Wettbewerbsbedingungen auf den Weltmärkten beigetragen hat, steckt sie seit einigen Jahren in einer ernsten Effektivitäts- und Legitimitätskrise. Dies ist bedauerlich, denn die WTO ist unabkömmlich für einen regelbasierten, fairen und transparenten Welthandel.

## Effektivitäts- und Legitimitätskrise der WTO

Unter dem Dach der WTO haben sich ihre mittlerweile 164 Mitglieder auf einen umfassenden Katalog von verbindlichen und diskriminierungsfreien Regeln geeinigt. Gemeinsames Ziel ist der weltweite Abbau von Handelshemmnissen. Mit ihren Transparenzmechanismen und der verbindlichen Streitbeilegung ist die Organisation das Rückgrat der internationalen Handelsordnung. Die Aufgaben der WTO können in vier Säulen unterteilt werden: Erstens bietet die WTO ein Forum für ihre Mitglieder, um Handelsbarrieren abzubauen und Regeln für den Welthandel zu setzen. Ihre zweite Aufgabe ist es, die Einhaltung der Regeln durch die Mitglieder zu überwachen. Sie verpflichtet ihre Mitglieder, nicht-tarifäre Handelshemmnisse und Subventionen zu melden. Zudem ist sie für die Schlichtung von Streitfällen zuständig. Schließlich ist sie eine Art Think Tank, der aktuelle Trends im Handel und der Handelspolitik erfasst, analysiert und veröffentlicht.

## Kaum Liberalisierungsfortschritte

Liberalisiert wird der Handel in der WTO zumeist in sogenannten Liberalisierungsrunden. Die letzte große Runde war die Doha-Entwicklungsrunde. Als diese 2001 begann, war die Hoffnung groß, dass der Handel – insbesondere der Agrar- und Dienstleistungshandel – weiter liberalisiert und neue Regeln geschaffen würden, um Protektionismus einzudämmen. Ungeachtet jahrelanger Verhandlungen hat die Doha-Runde abgesehen von dem Abkommen über Handelserleichterungen (Trade Facilitation Agreement, TFA) allerdings nur sehr wenig geliefert. Die EU und die USA sind nicht ganz unschuldig daran, dass der Agrarhandel nach wie vor durch hohe Zugangsbarrieren und Subventionen gekennzeichnet ist. Die Schwellen- und Entwicklungsländer zeigten ihrerseits wenig Bereitschaft, ihre Industriegüter- und Dienstleistungsmärkte stärker zu öffnen. Die Erwartungen an eine „echte Entwicklungsrunde“, um die Entwicklungsländer besser in die Weltwirtschaft zu integrieren, führten zu einer ideologischen Überfrachtung der Runde. Die Doha-Agenda war letztlich zu umfassend und zu ambitioniert. Die Priorisierung des Agrarhandels und fehlende sektorübergreifende Kompromisse führten immer wieder zu Blockaden in den Verhandlungen. Der Verhandlungsmodus (Single Undertaking: nichts ist beschlossen, bevor alles beschlossen ist) verhinderte lange die Realisierung von Teilerfolgen (wie beispielsweise bei Exportsubventionen im Agrarsektor). Die hohe Zahl der Mitglieder und das Konsensprinzip machten Kompromisse schließlich fast unmöglich.

Viele wichtige Bereiche des Welthandels sind daher noch gar nicht oder nur in geringem Umfang multilateral geregelt, z. B. Investitionen, Wettbewerb, öffentliches Auftragswesen und der digitale Handel. Mit der Ratifizierung des TFA haben sich die WTO-Mitglieder zwar weitestgehend vom „Single Undertaking“-Prinzip verabschiedet. Dennoch sind die wenigsten WTO-Mitglieder bereit, weitere multilaterale Vereinbarungen zu treffen. Dies schafft ein doppeltes Problem. Ohne entsprechendes Regelwerk, das dem Handel des 21. Jahrhunderts Rechnung trägt, kann die WTO neuen Protektionismus nicht angemessen in Schach halten. Darüber hinaus erfasste die Krise der ersten Säule der WTO mittlerweile auch die dritte Säule – die Streitbeilegung: Regierungen nutzten immer wieder den Streitschlichtungsmechanismus, um dort neues Recht zu schaffen, wo es bisher keine Regeln gab. Die WTO kennt eigentlich kein Präzedenzrecht; in der Praxis werden dennoch Streitschlichtungsentscheide bei der Lösung neuer Dispute herangezogen. Gerade die USA stören sich an dieser (aus ihrer Sicht) Mandatsüberschreitung des Streitschlichtungsmechanismus (insbesondere des Berufungsgremiums). Aus der Effektivitätskrise ist dementsprechend auch eine Legitimitätskrise der WTO geworden.

## Steigender Protektionismus

Zu Beginn der Wirtschafts- und Finanzkrise 2008 war die Sorge groß, dass sich eine Protektionismus-Spirale wie in der Großen Depression der 1930er Jahre wiederholen könnte. Die WTO trug maßgeblich dazu bei, dies abzuwenden. Die G20-Länder verständigten sich auf eine Stillstands- und Rückführungsverpflichtung. Zudem erfasst die WTO zusammen mit der Konferenz der Vereinten Nationen für Handel und Entwicklung (UNCTAD) seit Oktober 2008 regelmäßig neue protektionistische Maßnahmen ihrer Mitglieder. Dennoch konnte sie nicht verhindern, dass es seitdem zu einem schleichenden Protektionismus gekommen ist.

Von Oktober 2008 bis Oktober 2019 leiteten die WTO-Mitglieder insgesamt 1.654 handelsbeschränkende Maßnahmen – inklusive Zollerhöhungen, quantitativen Beschränkungen, strengeren Zollverfahren und neu ergriffenen Einfuhrzollsätzen und Ausfuhrzöllen (ohne Antidumping- und Antisubventionsuntersuchungen und -maßnahmen) – ein. Allein von Mai bis Oktober 2019 ergriffen die G20-Staaten restriktive Maßnahmen auf ein geschätztes Handelsvolumen von 460,4 Mrd. US-$. Das ist das zweithöchste Volumen seit Beginn der Berechnung im Jahr 2012. Vor zwei Perioden – also von Mai bis Oktober 2018 – war sogar ein Handelsvolumen von 480,9 Mrd. US-$ betroffen (WTO, 2008-2020).

Seit der Wahl Donald Trumps zum US-Präsidenten ist Protektionismus weltweit immer salonfähiger geworden. Anfang März 2018 beschlossen die USA die Einführung von Zöllen auf Stahl (25 %) und Aluminium (10 %), da Stahl- und Aluminiumimporte aus Sicht der Trump-Administration ein vermeintliches Sicherheitsrisiko für die USA darstellten (sogenannte Abschnitt-232-Zölle des Handelsgesetzes von 1962). Zahlreiche betroffene Länder antworteten ihrerseits mit Gegenmaßnahmen. Anfang 2020 weitete die Trump-Administration die 232-Zölle auf Stahl und Aluminium auf Produkte mit einem hohen Anteil dieser Metalle, wie etwa Nägel, Kabel und bestimmte Automobilteile, aus. Auch darauf gab es Gegenreaktionen im Ausland. Durch den Handelskonflikt zwischen den USA und China ist das Zollniveau deutlich gestiegen. Lag der durchschnittliche US-Zoll auf Importe aus China Ende 2017 noch bei 3,1 %, stieg dieser bis März 2020 auf 19,3 %. Die durchschnittliche Zollbelastung für US-Exporte nach China hat sich von Ende 2017 bis März 2020 von 8 % auf 20,3 % fast verdreifacht (Bown, 2020). Juristische Grundlage für die Zölle seitens der USA ist Abschnitt 301 des Handelsgesetzes von 1974.

Seit dem Beginn der Corona-Krise ist eine Vielzahl neuer Handelsbarrieren hinzugekommen – insbesondere Ausfuhrbeschränkungen. Diese betreffen vor allem persönliche Schutzausrüstung wie etwa Masken, Handschuhe und Schutzkleidung sowie Produktionsstoffe für Arzneimittel. Zudem werden solche Restriktionen für den Bereich medizinischer Geräte diskutiert. Bisher gibt es keine zentrale Erfassung der neuen Handelsbarrieren. Aufgrund oft unvollständiger Meldungen über neue Maßnahmen sind auch die Informationen der WTO nicht vollständig. Zurzeit haben 33 Länder und die EU 92 handels- und handelsverbundene Maßnahmen (einschließlich Exportrestriktionen und -verboten, außergewöhnlichen und temporären Kriterien, Aussetzung von Pflichtzertifizierung, Handelserleichterung) der WTO mitgeteilt (Stand 27. April 2020). Die tatsächliche Zahl der Exportrestriktionen ist wahrscheinlich deutlich höher, da einige Staaten, wie z. B. die USA oder Indien, ihre Exportrestriktionen nicht bei der WTO notifizieren (WTO, 2020a).

Das WTO-Sekretariat schätzt, dass bis zum 22. April 2020 „80 Länder und eigenständige Zollgebiete ... als Folge der COVID-19-Pandemie Ausfuhrverbote oder -beschränkungen eingeführt haben, darunter 46 WTO-Mitglieder (72, wenn man die EU-Mitgliedstaaten einzeln zählt) und acht Nicht-WTO-Mitglieder“. Der WTO zufolge betreffen die meisten Beschränkungen Güter, die von der Weltzollorganisation (WCO) als relevant für die Bekämpfung der Pandemie aufgeführt werden (COVID-19-Testkits, persönliche Schutzausrüstung, Thermometer, Desinfektionsmittel, medizinische Geräte, medizinische Verbrauchsgüter und Seife). Allerdings haben 17 Länder auch den Export von Lebensmitteln eingeschränkt (WTO, 2020a).

Dass die Dunkelziffer wahrscheinlich höher ausfällt, unterstrich auch ein Bericht von Global Trade Alert. Demnach wurden seit Jahresbeginn 2020 von 54 Regierungen insgesamt 46 neue Exportrestriktionen für medizinische Güter erlassen – 33 hiervon allein im März (Stand 21. März 2020) (Evenett, 2020). Mittlerweile erstrecken sich Ausfuhrbeschränkungen für medizinische Güter fast über den gesamten Globus. Während die ersten Ausfuhrbeschränkungen im Wesentlichen in asiatischen, arabischen und europäischen Ländern erlassen worden waren, haben mittlerweile auch die USA und zahlreiche lateinamerikanische und osteuropäische Länder solche Maßnahmen ergriffen (Stand 26. April 2020).

Hinzu kommen Verwerfungen in den weltweiten Wertschöpfungsketten und Produktionsnetzwerken, die durch die Unterscheidung zwischen vermeintlich systemrelevanten und nicht-systemrelevanten Produktionszweigen und Dienstleistungen in zahlreichen Ländern (auch bezeichnet als essenziell und non-essenziell) entstehen. In einigen Ländern müssen beispielweise Exporteure Nachweise erbringen, dass ausgeführte Waren im Abnehmerland Einsatz in systemrelevanten Produktionszweigen/Produkten finden werden. Eine solche Nachweispflicht wie beispielsweise für Kunststoffgranulat, Schrauben und Kabel, die dringend auch zur Herstellung von Beatmungsgeräten benötigt werden, verursacht einen weltweiten Flickenteppich unterschiedlicher Definitionen und Regelungen. Das dadurch entstehende rechtliche Chaos, verbunden mit erheblicher Intransparenz, führt zu einer enormen Rechtsunsicherheit für Unternehmen und einem massiven bürokratischen Aufwand, der den Handel weiter hemmt.

Die WTO hat kaum eine Handhabe, um gegen Exportbeschränkungen vorzugehen. Mit Artikel XI, Ziffer 1 haben sich die WTO-Mitglieder verpflichtet, keine mengenmäßigen Beschränkungen für den Export einzuführen. Ziffer 2 enthält jedoch eine umfassende Ausnahmeregelung: „Ausfuhrverbote oder Ausfuhrbeschränkungen, die während eines bestimmten Zeitraumes angewendet werden, um einer kritischen Lage vorzubeugen, die aus einem Mangel an Lebensmitteln oder anderen wichtigen Erzeugnissen für den ausführenden Vertragspartner entstehen könnte, oder um in einer solchen Lage Abhilfe zu schaffen“ sind gestattet. WTO-Mitglieder sind weder verpflichtet, die Maßnahmen bei der WTO zu melden, noch müssen sie nachweisen, dass ein Versorgungsenpass tatsächlich vorliegt.

## Weiterentwicklung des Regelwerks

Um das Regelwerk weiter zu entwickeln, setzen einige WTO-Mitglieder, darunter die EU, auf plurilaterale Abkommen. Zurzeit wird in der WTO über vier plurilaterale Initiativen verhandelt: E-Commerce, Dienstleistungen, Investitionen und kleine und mittlere Unternehmen. Im Mai 2019 legte die EU-Kommission einen umfangreichen Vorschlag für die plurilateralen Verhandlungen über den elektronischen Handel vor, der unter anderem elektronische Verträge und Authentifizierungen, Verbraucherschutz, grenzüberschreitende Datenflüsse und den Schutz personenbezogener Daten umfasst. Die EU unterstützt auch das multilaterale Moratorium, keine Zölle auf elektronische Übertragungen zu erheben (WTO, 2019).

Zudem hat die EU zusammen mit den USA und Japan im Rahmen der sogenannten trilateralen Initiative Vorschläge für den Umgang mit staatlichen Subventionen (insbesondere im Industriegütersektor) vorgelegt, vor allem durch eine Reform des WTO-Abkommens über Subventionen und Ausgleichsmaßnahmen (Office of the United States Trade Representatives, 2019). Beispielsweise spricht sich die trilaterale Initiative für einen breiteren Subventionsbegriff, die Erfassung von weiteren Arten von Subventionen, insbesondere auch solchen, die über Staatsunternehmen fließen, sowie strengere Meldepflichten und Sanktionsmechanismen aus. Die trilaterale Initiative drängt zudem auf Reformen der Regeln in den Bereichen Staatsunternehmen, Überkapazität, Marktverzerrung. Ziel ist es, einen fairen Wettbewerb gerade auch mit China herzustellen.

Die WTO hat schon jetzt eine Reihe plurilateraler Abkommen: Das Übereinkommen über das öffentliche Beschaffungswesen (GPA) und das Informationstechnologie-Abkommen (ITA I und II). Darüber hinaus hat eine Reihe von WTO-Staaten 2013 begonnen, ein neues Abkommen über den Dienstleistungshandel (Trade in Services Agreement, TiSA) zu verhandeln. Diese Verhandlungen und die Verhandlungen über ein plurilaterales Abkommen zur Beseitigung von Zöllen auf sogenannte Umweltgüter liegen derzeit auf Eis – unter anderem, weil sie bislang durch die aktuelle US-Regierung nicht angepackt werden. Zudem drohten die USA wiederholt mit einem Austritt aus dem GPA.

Plurilaterale Abkommen unter der WTO sind umstritten. Gerade die Schwellen- und Entwicklungsländer, allen voran Indien, stehen solchen Initiativen, wie für den digitalen Handel, kritisch gegenüber. Auch wenn sich viele von ihnen den genannten Initiativen nicht angeschlossen haben, fürchten sie, dass der Druck über die Märkte so stark steigen könnte, dass sie einem Abkommen zustimmen müssen, ohne die Regeln mit gesetzt zu haben. Zudem kritisieren sie, dass diese Initiativen den Handelsspielraum gerade für Entwicklungs- und Schwellenländer zu stark einschränkten. Hinzu kommt, dass eine zentrale Frage noch nicht abschließend geklärt wurde: Ab wann kann ein solches Abkommen in Kraft treten? Damit ein plurilaterales Sektorabkommen innerhalb der WTO geschaffen werden kann, bedarf es einer kritischen Masse an WTO-Mitgliedern, die das Abkommen unterzeichnen. Dieses Kriterium ist dann erreicht, wenn die Teilnehmer ungefähr 90 % des Welthandels für den entsprechenden Sektor abdecken. Dieses Kriterium hat in der Vergangenheit neue Sektorabkommen verhindert, da die großen Schwellenländer diese ablehnen. Ob dieses Kriterium auch für plurilaterale Regelungsabkommen wie ein Abkommen über Subventionen gilt, ist ungeklärt. Ebenso ungeklärt ist die Frage, wann alle WTO-Mitglieder über die unbedingte Meistbegünstigung (MFN) der WTO in den Genuss von Marktöffnungen unter einer solchen Übereinkunft kommen und wann diese nur für die Mitglieder gelten können, das Abkommen also präferenziell (diskriminierend) sein darf.

## Fehlende Notifizierung

Die WTO wendet sich mit verschiedenen Instrumenten gegen protektionistische Maßnahmen und kontrolliert damit die Einhaltung ihrer Handelsregeln. Dazu gehören insbesondere der Mechanismus zur Überprüfung der Handelspolitik (Trade Policy Review Mechanism, TPRM), die Berichte des WTO-Generaldirektors sowie das integrierte Portal der WTO zu Handelsmaßnahmen (Integrated Trade Intelligence Portal, I-TIP). Die genannten Instrumente führten bereits zu mehr Transparenz, da sie aufzeigen, wo Staaten protektionistische Maßnahmen ergriffen haben. Wirkliche Konsequenzen gibt es für die Staaten aber nicht. Es handelt sich eher um „naming and shaming"-Mechanismen.

Die Notifizierungspflichten bei der WTO sind nicht nur schwach, viele Länder erfüllen ihre Meldepflichten nur unzureichend oder gar nicht. Die EU legte daher zusammen mit den USA und mehreren anderen WTO-Mitgliedern Mitte 2019 einen Entwurf für strengere Notifizierungspflichten vor. Diskutiert wird nicht nur, wie die Anreize für WTO-Länder verbessert werden können, handelsbezogene Maßnahmen bei der WTO zu melden. Beispielsweise beklagen kleinere Entwicklungsländer oftmals fehlende eigene Kapazitäten. Der Vorschlag enthält zudem einen Sanktionsmechanismus. Daher ist er gerade bei den Schwellen- und Entwicklungsländern auf große Ablehnung gestoßen.

## Defizite in der Streitschlichtung

Besonders brisant ist die Krise des Streitschlichtungsmechanismus (Dispute Settlement Mechanism, DSM). Im DSM können WTO-Mitglieder im Fall eines potenziellen Verstoßes gegen WTO-Regeln gegen andere Mitglieder vorgehen. Beim DSM wird durch bilaterale Konsultationen und durch die Einsetzung von Panels (erste Instanz) zunächst versucht, eine einvernehmliche Lösung zu finden, in zweiter Instanz kann eine Partei in Berufung gehen. Vor dem sogenannten Berufungsgremium (Appellate Body) wird die Panelentscheidung nochmals überprüft. Am Ende können Handelssanktionen stehen, wenn das betreffende Land die regelwidrigen Maßnahmen nicht fristgerecht korrigiert.

Mit 39 neuen Handelskonflikten stieg die Zahl der Dispute 2018 deutlich an. 2019 wurden 19 neue Beschwerden bei der WTO eingereicht (WTO, 2020b). Viele dieser Handelskonflikte sind hoch politisch. Aktuell blockieren die USA die Ernennung neuer Mitglieder für das Berufungsgremium. Mittlerweile hat der normalerweise siebenköpfige Appellate Body deshalb nur noch ein Mitglied. Da drei Mitglieder notwendig sind, um über einen Fall zu befinden, ist das Gremium seit Mitte Dezember 2019 funktionsunfähig. Sollte ein WTO-Mitglied gegen einen Panelbericht Berufung einlegen, würde dies „in eine rechtliche Leere" führen, da das Berufungsgremium nicht mehr funktioniert. Entsprechend kann keine endgültige, verbindliche und durchsetzbare Entscheidung getroffen werden. Zu den Kritikpunkten zählen unter anderem die lange Dauer der Bearbeitung der Streitfälle, die häufig die gesetzten Fristen überschreitet. Zudem lehnen die USA ab, dass Mitglieder des Berufungsgremiums laufende Fälle nach ihrem Austritt aus dem Gremium weiter behandeln. Schließlich unterstellen die USA dem Berufungsgremiun, sein Mandat zu überschreiten. Auf Druck der USA wurde im Dezember 2019 zudem das WTO-Budget für die Streitschlichtung im Jahr 2020 massiv zurückgeschnitten.

Die EU gehört zu den aktivsten Nutzern des DSM. Seit Gründung der WTO 1995 bis Ende 2019 reichte die EU 104 Mal Klage ein. China trat seit seinem Beitritt zur WTO im Jahr 2001 in 21 Fällen als Kläger auf. Die USA reichten 124 Mal Klage ein (21 % der gesamten 593 Streitschlichtungsfälle). Um den Streitbeilegungsmechanismus nicht vollständig lahmzulegen, schlug die Europäische Kommission einen Ad-hoc-Streitbeilegungsmechanismus vor. Dieser soll so lange zum Einsatz kommen, wie die WTO-Berufungsinstanz blockiert wird. Am 21. Oktober 2019 folgte eine Vereinbarung zwischen der EU und Norwegen. Ende März 2020 mündeten diese Bemühungen im Multi-Party Interim Appeal Arbitration Arrangement (MPIA). MPIA gilt zunächst für die EU und 15 weitere Staaten, ist jedoch für alle interessierten Staaten offen. MPIA wird die Berufungsinstanz der WTO in der Streitbeilegung ersetzen, bis eine multilaterale Lösung unter dem Dach der WTO gefunden wird. Zudem wurde eine Vielzahl an Vorschlägen für die Reform des Streitschlichtungsmechanismus vorgelegt. Ein Kompromiss ist jedoch nicht in Sicht.

## Differenzierung nach Entwicklungsstatus

Ein weiterer Streitpunkt ist die Klassifizierung von Ländern nach Entwicklungsstatus in der WTO. Entwicklungsländer genießen eine besondere und differenzierte Behandlung innerhalb der WTO. Das heißt beispielsweise, dass sie ihre Märkte nicht in demselben Maße öffnen müssen wie Industrieländer. Kriterien, die zwischen Entwicklungs- und Industrieländern unterscheiden, hat die WTO allerdings nicht. Vielmehr bestimmen die Mitglieder ihren Entwicklungsstatus selbst. Folglich werden große Schwellenländer wie China (aber auch Brasilien, Argentinien, Indien, Russland und viele andere) immer noch als Entwicklungsländer geführt. Im September 2018 sprachen sich die USA zusammen mit der EU und Japan dafür aus, dass wirtschaftlich weit entwickelte Schwellenländer stärker in der WTO in die Pflicht genommen werden. Zudem schlugen die USA strenge Kriterien für die Bestimmung des Entwicklungsstatus vor. Diese gehen allerdings den meisten WTO-Mitgliedern deutlich zu weit.

## Ausblick: Wie kann es mit der WTO weitergehen?

Die Krise der WTO hat viele Gründe. Der Konsens für eine liberale Handelsordnung und der Glaube, dass Handel Wirtschaftswachstum und Entwicklung fördert, ist erodiert. Die Interessen der WTO-Mitglieder sind sehr heterogen; das Konsensprinzip erschwert die Entscheidungsfindung. Die WTO-Regeln tragen schon lange nicht mehr den Charakteristika des modernen Handels Rechnung. Die WTO-Mitglieder haben seit Jahren die Priorität auf den Abschluss bilateraler Handelsabkommen gelegt. Jüngst kommt noch der Systemwettbewerb zwischen den USA und China hinzu (Felbermayr, 2020).

Die Corona-Krise zeigt jedoch, wie wichtig die WTO ist. Um den Stillstand in der Organisation zu beenden und die Lähmung des DSM zu durchbrechen, sind Reformmaßnahmen in allen vier Säulen der WTO notwendig. Die EU und andere Mitglieder haben – teils gemeinsam – verschiedene Reformvorschläge vorgebracht. Die WTO ist eine intergovernmentale Organisation. Der Handlungsspielraum des Sekretariats und des Generaldirektors ist limitiert. Auch wenn die für den Sommer 2020 geplante Ministerkonferenz auf das Jahr 2021 verschoben wurde, ist es dringend an der Zeit, dass sich die WTO-Mitglieder ernsthaft zusammensetzen, um sich konstruktiv auf einen Reformweg zu verständigen. Dabei muss es sowohl um die Rolle des Sekretariates (eine Stärkung), die Entscheidungsfindung (auch in den Ausschüssen), eine Weiterentwicklung des Regelwerkes, eine Stärkung der Notifizierungspflicht sowie die Stärkung des DSM gehen. Nur so kann die multilaterale Organisation auch in Zukunft die Hüterin des Welthandels bleiben. Und nur so kann verhindert werden, dass der Protektionismus weiter um sich greift.


Eckart von Unger, Dipl.-Volkswirt, ist stellvertretender Leiter, und Katherine Tepper, M. A., ist Senior Referentin in der Abteilung Außenwirtschaftspolitik des BDI in Berlin.


Literatur

Bown, C. (2020), US-China Trade War Tariffs: An Up-to-Date Chart, Peterson Institute for International Economics, https://www.piie.com/research/piie-charts/us-china-trade-war-tariffs-date-chart (13. Mai 2020).
Evenett, S. (2020), Tackling COVID-19 Together, März 2020, https://www.wita.org/atp-research/tackling-covid-19-together-the-trade-policy-dimension/ (13. Mai 2020).
Felbermayr, G. (2019), 25 Jahre WTO — Ursachen des Zerfalls und Reformvorschläge für die Zukunft, Kiel Focus, https://www.ifw-kiel.de/de/publikationen/kiel-focus/2019/25-jahre-wto-ursachen-des-zer-falls-und-reformvorschlaege-fuer-die-zukunft-0/ (13. Mai 2020).
Internationaler Währungsfonds (IMF) (2020a), World Economic Outlook: The Great Lockdown, April 2020, https://www.imf.org/en/Publications/WEO/Issues/2020/04/14/weo-april-2020 (13. Mai 2020).
Internationaler Währungsfonds (IMF) (2020b), World Economic Outlook: Tentative Stabilization, Sluggish Recovery?, Januar 2020, https://www.imf.org/en/Publications/WEO/Issues/2020/01/20/weo-update-january2020 (13. Mai 2020).
Obstfeld, M. und A. S. Posen (Hrsg.) (2020), How the G20 Can Hasten Recovery from COVID-19, Peterson Institute for International Economics, PIIE Briefing, 20–1, April 2020, https://www.piie.com/publications/piie-briefings/how-g20-can-hasten-recovery-covid-19 (13. Mai 2020).
Office of the United States Trade Representative (2020), Joint Statement of the Trilateral Meeting of the Trade Ministers of Japan, the United States and the European Union, https://ustr.gov/about-us/policy-offices/press-office/press-releases/2020/january/joint-statement-tri-lateral-meeting-trade-ministers-japan-united-states-and-european-union (13. Mai 2020).
Welthandelsorganisation (WTO) (2019), Joint Statement on Electronic Commerce — EU Proposal for WTO Disciplines and Commitments relating to Electronic Commerce, https://trade.ec.europa.eu/doclib/docs/2019/may/tradoc_157880.pdf (13. Mai 2020).
Welthandelsorganisation (WTO) (2020a), COVID-19 and World Trade, https://www.wto.org/english/tratop_e/covid19_e/covid19_e.htm (13. Mai 2020).
Welthandelsorganisation (WTO) (2020b), Disputes by Member, https://www.wto.org/english/tratop_e/dispu_e/dispu_by_country_e.htm#top (13. Mai 2020).
Welthandelsorganisation (WTO) (2020c), Trade Set to Plunge as COVID-19 Pandemic Upends Global Economy, https://www.wto.org/english/news_e/pres20_e/pr855_e.htm (13. Mai 2020).
Welthandelsorganisation (WTO) (2008–2020), Trade Monitoring, https://www.wto.org/english/tratop_e/tpr_e/trade_monitoring_e.htm (13. Mai 2020).
